# On the Distribution of the Nerves of the Dental Pulp

**Published:** 1912-07-15

**Authors:** J. Howard Mummery


### ON THE DISTRIBUTION OF THE NERVES OF THE DENTAL PULP.

BY J. HOWARD MUMMERY.

Communicated to the Royal Society by Professor F. Symington, F.R.S., February 1912.

The mode of innervation of the dentine of the human tooth has long been a matter of controversy; while clinical evidence is strongly in favor of a nerve supply to this tissue, the difficulties met with in tracing nerve-fibres in such a difficult substance to examine as the dentine has been very considerable hindrance to the investigation. It has been very difficult to account for the passage of such very acute sensation from the periphery of the dentine in the absence of nerve-fibres in that situation, and I have long felt, with others that sensation in the tooth would be found to be conducted by nerve-fibres, as in other tissues of the body. As long ago as 1891 I made preparations which appeared to show that nerve fibres from the pulp entered the dentine, but, by the iron and tannin impregnation process I then employed, could not satisfactorily demonstrate it.

During the last year I have, I think, with several methods of preparation, been successful in making it fully evident that the dentine is richly supplied with nerves from the pulp, which do not terminate, as has been hitherto generally supposed, at the inner margin of the dentine, but enter the tubules of that tissue and traverse them to their peripheral terminations at the enamel and cementum margins.

The bundles of medullated fibres which enter the tooth at the apical foramen traverse the pulp in more or less parallel lines, running in most cases in company with the blood-vessels, They send off numerous side branches, which at the periphery at the periphery of the pulp lose their medullary sheath, the axis cylinders spreading out into a mass of neurofibrils which enter into a more or less dense plexus beneath the odontoblast layer of cells. From this plexus, known as the plexus of Raschkow, fine neurofibrils pass between and around the odontoblasts, enclosing them in a fine meshwork, and enter into a narrow plexus at the inner margin of the dentine. This has usually been described as the modé of termination of the nerve-fibres of the pulp, but fibres can be seen arising from this plexus, which might be better termed the "marginal plexus," and passing into the dentinal tubes.

These neurofibrils pass into the dentine in great abundance and seem to be equally distributed in the coronal portion and considerably below the neck of the tooth, but become more and more scattered as they approach the apex of the root. This is especially well shown by Ramon y Cajal's silver nitrate process. The most successful preparations have been those prepared with silver nitrate and with gold chloride, but the erratic manner in which these substances select the tissues is well known, and it is only here and there among some hundreds of sections that a thoroughly successful impregnation is found.

In this investigation I have made use of fresh calcified teeth ground on a lathe, of teeth decalcified with nitric acid, and with formic acid, and of calcified teeth ground on a stone after impregnation with balsam by the Weil process. This latter method appears to me superior to all others, although a very tedious and troublesome one to carry out successfully; good preparations fully repay the trouble; there appears to be no shrinking of the cells, such as occurs in specimens decalcified with acids, and the matrix of the dentine is not stained. The staining of the matrix in decalcified specimens greatly interferes with the clear observation of the contents of the tubules, the minute longitudinal striation seen in the matrix especially in silver preparations, giving rise to very deceptive appearances. In a well impregnated balsam preparation the contents of the tubules are stained with the silver, the surrounding matrix remaining quite clear and unstained.

I have also procured some very successful slides by staining a small piece of a calcified tooth in bulk with a nuclear stain, passing it through the nitrate of silver process, and then decalcifying with formic acid.

In the substance of the dentine in well-impregnated preparations fine beaded fibres can be traced in the tubules, and in the majority of cases there appear to be two fibres in each tubule which can be traced in many preparations to the inner margins of the enamel and cementum.—(Abstract of the *Proceedings of the Royal Society*, B. Vol. 85.)

